# Standalone Regulatory Agreements for Product-Development Collaborations in the Medical Products Industry

**DOI:** 10.1007/s43441-024-00646-1

**Published:** 2024-05-31

**Authors:** Mary E Wilhelm, Nancy Pire-Smerkanich, Frances J Richmond

**Affiliations:** 1 Ascendis Pharma, 1000 Page Mill Road, Palo Alto, CA USA; 2 Department of Regulatory and Quality Sciences and DK Kim International Center for Regulatory Science, USC Mann School of Pharmacy, University of Southern California, Los Angeles, CA USA

**Keywords:** Regulatory templates, Drug development, Device development, Legal agreements, Regulatory agency, Outsourcing

## Abstract

**Background:**

Medical-product companies often outsource research and manufacturing needs to contracting or partnering organizations but then must manage a challenging patchwork of regulatory activities. A standalone regulatory agreement could clarify the relationships and responsibilities between companies working jointly on a single regulated product. This study explored the need for and current use of standalone regulatory agreements.

**Methods:**

A survey instrument was developed using an implementation framework and disseminated to mid- to senior-level employees and consultants for sponsor and vendor companies in the medical products sector.

**Results:**

Of 294 respondents, about half, primarily from companies with more than 200 employees, were familiar with standalone regulatory agreements, and half of this subgroup had moved forward to implement them. Such agreements were considered beneficial to clarify regulatory roles and responsibilities, standardize regulatory expectations between the companies, and stimulate earlier discussion about joint regulatory strategies. However, the development of regulatory agreements appears challenged by the fact that such agreements are not required by regulatory agencies overseeing medical products and have no standardized templates, agency or industry guidance. Respondents whose organizations do not now use regulatory agreements either had not considered or did not see a need for a standalone agreement.

**Conclusions:**

Standalone regulatory agreements are becoming more common but are not yet implemented fully by most companies. Their usefulness and content appeared to depend upon the type of partner, the complexity of the relationship and the availability of internal expertise and support.

**Supplementary Information:**

The online version contains supplementary material available at 10.1007/s43441-024-00646-1.

## Introduction

Pharmaceutical development over the last few decades has evolved to take advantage of external contractors that conduct specialized activities [1–3]. Partnering with storage and distribution suppliers, Contract Development and Manufacturing Organizations (CDMOs), and Contract Research Organizations (CROs) can decrease clinical and production costs and reduce the need to hire and train new personnel. The importance of such relationships is reflected in the growth of specialist contractors that in clinical research alone now contribute to what has become a $40 Billion global industry [4].

Beginning interactions with CDMOs and CROs are typically transactional. The deliverables are usually determined before the relationship is established and the contracted agreements reflect the nature of an often-narrow service. With time, however, companies have become comfortable with contracts for a variety of more complicated relationships. These might include, for example, Quality Agreements governing the product’s ingredients and containers, Technical Agreements that detail the quality and testing of the product, and Supply Chain Agreements that define terms for pricing and payment, minimum and maximum purchase quantities, delivery lead times, product return policies, and liabilities when materials are lost [5]. Further, Clinical Trial Agreements typically define the responsibilities of the sponsor, CRO and clinical site, terms governing publication and intellectual property, and arrangements for record keeping, inspection, indemnification, and insurance [6]. To manage these types of partnerships, companies may alternatively use a Master Service Agreement (MSA) that “consolidates separate but related agreements between the same signing parties” [7]. The MSA will define more expectations- confidentiality, delivery requirements, dispute resolution, intellectual property rights and payment terms, for example- that each partner must satisfy to fulfill the contract.

However, more recently, the interactions between a sponsor and its partners have changed in ways that do not lend themselves to simpler contracts. Relationships through unrestricted grants with centers created for drug discovery, or partnerships with specialized CROs, academia and biotech companies, for example, may be less clearly defined, may involve risk-sharing and may change as new ideas and specialized technologies are introduced to address the increased complexities of drugs in development [2, 3, 8, 9]. One particular concern is the way that the two organizations will contribute to the regulatory deliverables and filings related to that product during the time of the partnership, which often extends over many years. As the relationship evolves from early development to product approval, changing events and circumstances can affect the expectations of partners over time. Thus, their roles with respect to regulatory filings can be more difficult to capture in simple transactional contracts or high-level business agreements because they fail to cover important areas of responsibility and expectations.

Standalone regulatory agreements have the potential to address more directly the regulatory expectations between partners in joint ventures and mergers of some types. Some expected areas could include the preparation of submission documents, review of nonclinical and clinical research reports, attendance at health agency meetings, review of product labeling, advertising and promotion, and ownership of original submission documents and records. However, the use of regulatory agreements has not been described in any detail by current literature. We therefore have no clear picture of the extent to which regulatory agreements have been introduced and what regulatory requirements might be most important to address. Some companies may rely on other existing agreements to meet this need, but we do not know the extent to which such an approach has been used. We also have little insight into the previous experiences of companies in partnership agreements and whether they believe that a more comprehensive regulatory agreement would have been helpful. A primary goal of this research is to identify current views and experiences of individuals in companies conducting joint development programs that require some form of regulatory sharing. From this information, we discuss the potential role of stand-alone regulatory agreements and propose a framework for their content.

## Materials and Methods

This study explored the views and experiences of mid- to senior-level employees at sponsor and vendor companies in the medical products sector whose experiences in drug development encompassed activities in regulatory, quality, clinical, or product development. Included also were consultants and individuals experienced with legal, financial, business development or alliance management activities during product development. Prior to this study, we explored the available literature systematically to identify whether any previous guidelines regarding regulatory agreements were available [10]. Further, no public database was found to identify systematically individuals who would be appropriate respondents. To assure a broad representation from companies of different sizes, participants were identified by title through LinkedIn networks, personal referrals, social networking platforms and professional associations. Respondents were considered to meet inclusion criteria if they worked at medical product companies in regulatory, clinical, or quality roles and had recent or current experience with the conduct of joint development programs. Interested respondents were encouraged to nominate others to participate.

A self-administered survey instrument was built with reference to an implementation framework described by Fixsen and coworkers [11, 12], which divided implementation into Exploration, Installation, Initial Implementation and Final Implementation [13]. Its 42 questions were configured using multiple-choice, scaled, matrix, rank-ordered and open-text formats. A brief introduction defined what is meant by a regulatory agreement and asked respondents to provide answers based on their experiences with one recent company in which they worked. Questions were designed to solicit input on the current use or possible need for regulatory agreements between partnering companies, including vendor companies such as a CDMO or CRO, or a company and academia (Appendix A). Three skip-logic branches were used to hide more detailed questions from participants whose answers to preliminary questions suggested little experience in a specialized area. The first branch divided participants who had heard of standalone regulatory agreements from those who had not. The next branch separated respondents who did not have collaborations with other companies or academic institutions from respondents who had investigated the use of standalone regulatory agreements from those who had not. A third branch separated respondents who had implemented or planned to implement regulatory agreements from those who did not move forward or had not investigated these agreements. A focus group of 8 participants from industry and academia critiqued the survey, which was then revised to address their suggestions.

The survey was deployed anonymously to 1194 potential participants using the web-based survey platform, Qualtrics (http://www.qualtrics.com/); 294 started the survey and 244 completed at least one question for a response rate of 20% and a completion rate of 83%. An additional 50 participants accessed the survey using an anonymous link and completed at least one question for the combined total of 294 respondents. Respondents were allowed to skip questions so the number of respondents answering each question varied and is shown for each set of answers. Cumulative results were analyzed using descriptive statistics and cross-tabulations. Open text and comment fields were analyzed to identify themes or common elements. One question used a rank order format so that respondents could rank their preferences. A rank score was calculated by applying a weight to each response. Because this question used a 5-point scale, the number of responses where the option was ranked as 1 (or most likely) was multiplied by 5, as 2 was multiplied by 4, as 3 by 3, as 4 by 2, and as 5 by 1. The total weighted score for each option was summed and divided by the number of respondents, to get the final rank option, sorted from highest (1) to lowest (5).

## Results

### Study Dissemination

Respondents ranged in experience and functional role; three-quarters were at or above the Director level and represented companies across a spectrum of sizes (Table 1). Most worked for sponsor companies (76%). The others were in consultancies (15%), clinical support organizations (6%), law firms or in-house counsel roles (1%) or other industry-related organizations (2%). 65% dealt with pharmaceuticals, 57% with biologics/biotechnology products, 33% with medical devices/diagnostics, and 32% with combination products. Many had experience with more than one product type, as reflected by total percentages much higher than 100%. 3% worked in other areas, which they identified as cell or gene therapies, cosmetics, dietary supplements, food additives, industrial chemical, and veterinary biologicals.

### Companies with Regulatory Agreements

**Fig. 1:**
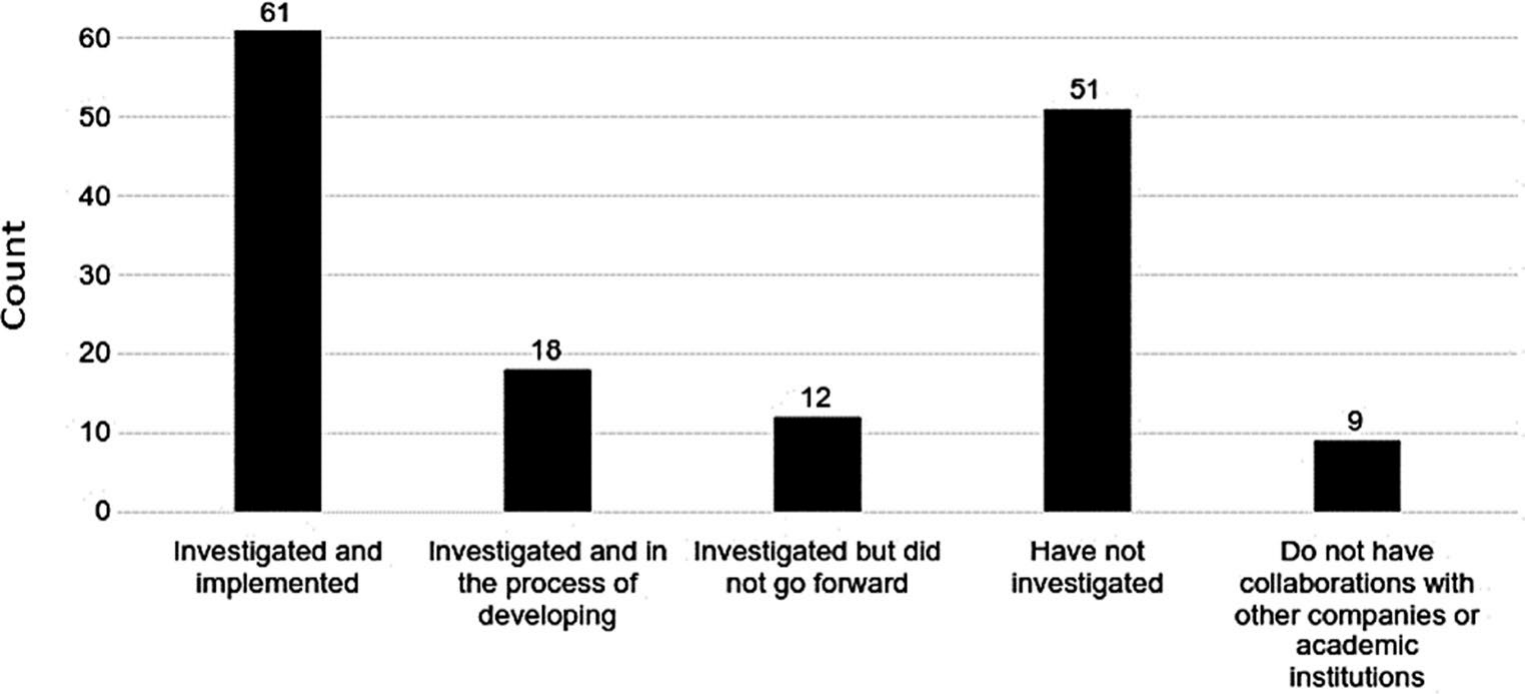
Use of Regulatory Agreements with Business or Academic Partners. Please think about your collaboration projects with other pharmaceutical, biotechnology, or device companies or with an academic institute. Has your company investigated the use of standalone regulatory agreements for any of these collaborations? (number of responses = 151 of 152 respondents presented with this question)

About half of the respondents had previously heard of standalone regulatory agreements developed as a separate document (yes: 54%; no: 46%) (Table 1). 40% of respondents who had heard about regulatory agreements had investigated and implemented the use of standalone regulatory agreements, whereas 12% were in the process of developing them and 34% had not investigated their use (Fig. 1). In addition, 8% of this group had investigated these types of agreements but did not go forward. Only 6% were not involved in collaborations with other companies or academic institutions.

**Table 1:** Awareness of Regulatory Agreements. Cross-tabulation of company size, respondent title, and product type with respect to awareness of the use of regulatory agreements (number of responses = 283 of 294 respondents presented with this question)

		Have you heard of using standalone regulatory agreements developed formally as a separate document … during product development and commercialization?		
Question	Choice	Total	Yes	No
	Total Employee Count	283	152	131
Which statement best describes the size of your overall organization?	< 200	117 (41%)	55 (36%)^1^	62 (47%)^2^
	201 - 2000	86 (30%)	47 (31%)	39 (30%)
	2001 - 50,000	48 (17%)	33 (22%)	15 (12%)
	>50,000	32 (11%)	17 (11%)	15 (12%)
Which title is most closely aligned with your current responsibilities?	Vice President / C-Suite	95 (34%)	51 (34%)	44 (34%)
	Director	120 (42%)	67 (44%)	53 (41%)
	Manager	20 (7%)	10 (7%)	10 (8%)
	Specialist / Associate	12 (4%)	4 (3%)	8 (6%)
	Attorney / Legal Counsel	5 (2%)	3 (2%)	2 (2%)
	Consultant	24 (9%)	13 (9%)	11 (8%)
	Other	7 (3%)	4 (3%)	3 (2%)
With which product types do you work? (choose all that apply)	Biologics / Biotechnology	162 (57%)	88 (58%)	74 (57%)
	Pharmaceuticals	185 (65%)	96 (63%)	89 (68%)
	Combination Products	91 (32%)	47 (31%)	44 (34%)
	Medical Devices / Diagnostics	94 (33%)	57 (38%)	37 (28%)
	Other	9 (3%)	5 (3%)	4 (3%)

^1^Percent is of the number of respondents answering yes

^2^Percent is of the number of respondents answering no

Respondents structured a variety of relationships when they worked with other pharmaceutical, biologics/biotechnological, medical device, support-service and vendor companies, academic institutions, and consultancies. Most commonly, their partnerships were governed by formal agreements (55-87%) (Table 2). Preferred partner/vendor contracts governed less than 8% of relationships with their consultants and pharmaceutical, biotechnology and academic organizations but were somewhat more frequent for partnerships with device companies (11%) and support-service/vendor organizations (23%). Informal agreements and research grants were rare (< 5%) amongst commercial organizations. However, about 10% of academic agreements were informal and a further 19% were research grants.

**Table 2:** Structure of Company Relationships With Partners. What type of relationships does your company have with your partners? (choose all that apply) (number of responses = 286 of 294 respondents presented with this question)

Company Type	Formal agreements	Preferred partner/ vendor	Informal means such as past work relationships	Research grants	None	Total
Pharmaceutical Companies	201 (78%^1^)	11 (4%)	5 (2%)	2 (1%)	40 (15%)	259
Biologics / Biotechnology Companies	157 (67%)	16 (7%)	4 (2%)	0 (0%)	57 (24%)	234
Medical Device Companies	113 (55%)	23 (11%)	5 (2%)	0 (0%)	65 (32%)	206
Vendors (CDMO / CRO)	180 (72%)	57 (23%)	5 (2%)	0 (0%)	9 (4%)	251
Academic Institutions	117 (56%)	7 (3%)	21 (10%)	40 (19%)	25 (12%)	210
Consultants	216 (87%)	13 (5%)	8 (3%)	1 (0%^2^)	9 (4%)	247
Other	7 (25%)	1 (4%)	1 (4%)	1 (4%)	18 (64%)	28

^1^Percent is the number of respondents by the total for the row

^2^value of zero due to rounding

Companies relied on different resources when first developing a regulatory agreement. Almost all identified that internal company experience/expertise was useful (91%) and most that the structure/contents of other contracts or scope of other partnerships were useful (85% and 77% respectively). Government documents and external consultants were regarded as useful by 56% and 54% of respondents respectively, but as not useful by 23%.

**Fig. 2:**
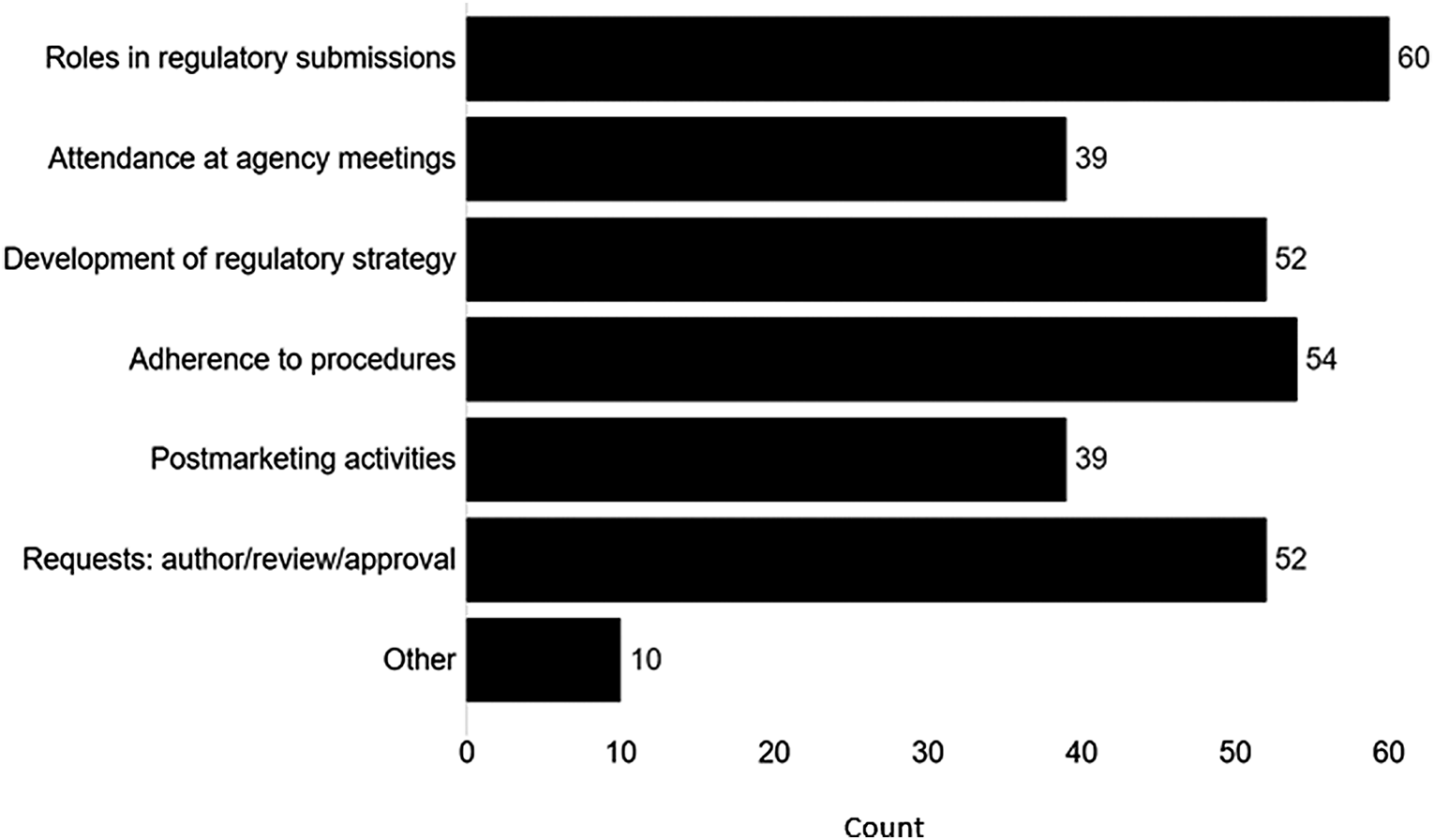
Development Elements Included in Initial Regulatory Agreements. What development elements were included in the initial regulatory agreement? (choose all that apply) (number of responses = 70 from 79 respondents presented with this question)

Respondents with regulatory agreements were asked if 6 development elements related to regulatory strategy, reviews and logistics were included in these initial agreements (Fig. 2). Respondents selected these choices in the following order: roles in regulatory submissions (86%); adherence to regulatory procedures (77%), development of regulatory strategy (74%); regulatory requests authoring, review, and approval (74%); attendance at agency meetings (56%); and postmarketing activities (56%). 14% identified “other” elements with responses related to documentation, compliance, responsibility, or communication.

**Fig. 3:**
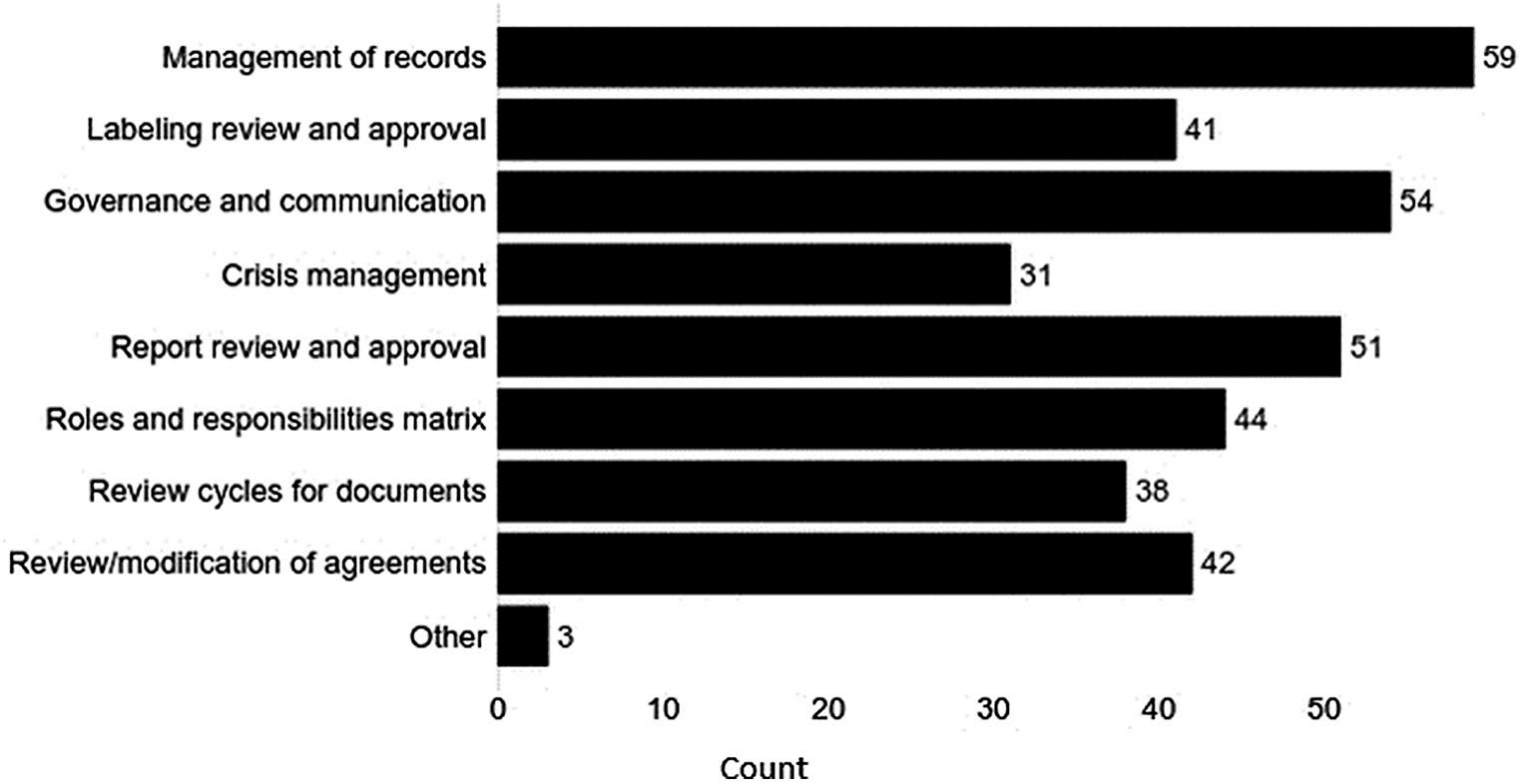
Review and Logistics Elements Included in Initial Regulatory Agreements. What review or logistics elements were included in the initial regulatory agreement? (choose all that apply) (number of responses = 70 from 79 respondents presented with this question)

The respondents were also asked if 4 elements related to regulatory reviews and 4 elements related to logistics were included in those initial agreements (Fig. 3). A majority chose all elements related to regulatory review: report review and approval (73%); timing for review/modification of agreement (60%); review and approval of labeling (59%); number and timing of review cycles for documents (54%). The 4 logistical elements were chosen by many: management of records (84%); governance and communication processes (77%); development of RACI matrices defining roles for Responsible, Accountable, Consulted, and Informed parties (63%); and roles and responsibilities to manage crisis (44%). Three respondents (4%) selected “other”, adding safety reporting and notifications of audits, inspections, and adverse events.

When asked about additional elements that they would include in the next revision of their company’s regulatory agreement, 35 comments were received from 73 respondents presented with this question. Substantive comments were grouped into 4 themes- compliance; roles and responsibilities; document review; and processes (Table 3).

**Table 3:** Regulatory Agreement Elements to Include in the Revised Agreement. What elements, if any, would you include in the next revision of the regulatory agreement that you did not have in the initial regulatory agreement (number of responses = 35 from 73 respondents presented with this question)

Them	Comment
Compliance	• More shifting liability clauses • Clearer milestone agreements • Crisis management (n = 2) • Penalties for non compliance • Consequences for lack of performance • Database management and compliance
Roles and Responsibilities	• Attendance AND roles/responsibilities for agency interactions (Written, telephone, and face to face) • More specific detail of what each company is responsible for even though the document is currently a template format to allow for more explicit documentation of who does what, when, how, and any responsibilities of the other party for review, approval, editing, etc • Specific roles of individuals • Sign off by wider range of functional areas of both parties. • Specific roles and responsibilities of consultants • Matrix defining RACI • In general, the partnership template we utilize provide consistency. Some partnerships may be different (i.e. EU right only, not including US, or specific regions) so outlining of regional specific RA requirement, including R/R in each region will be key to avoid future confusions • More assignment of roles and crisis arrangements. Review period. • More information on project teams and levels of involvement for submissions • A commitment from the supplier that they will maintain qualified and knowledgeable regulatory and quality resources to support the commitments within this agreement, in case the current resources are fired, laid off, or leave the company.
Document Review	• Timing for review/modification • Process/cycles for drafting and review of agreement; continuous maintenance of agreement. • Turnaround time for documents from CRO
Process	• Process workflow diagram for collecting, reviewing, approving, storing and submission. • Audits • Regulatory due diligence • Key performance indicators • info sharing • Assumptions for the agreement

### Companies without Regulatory Agreements

**Fig. 4:**
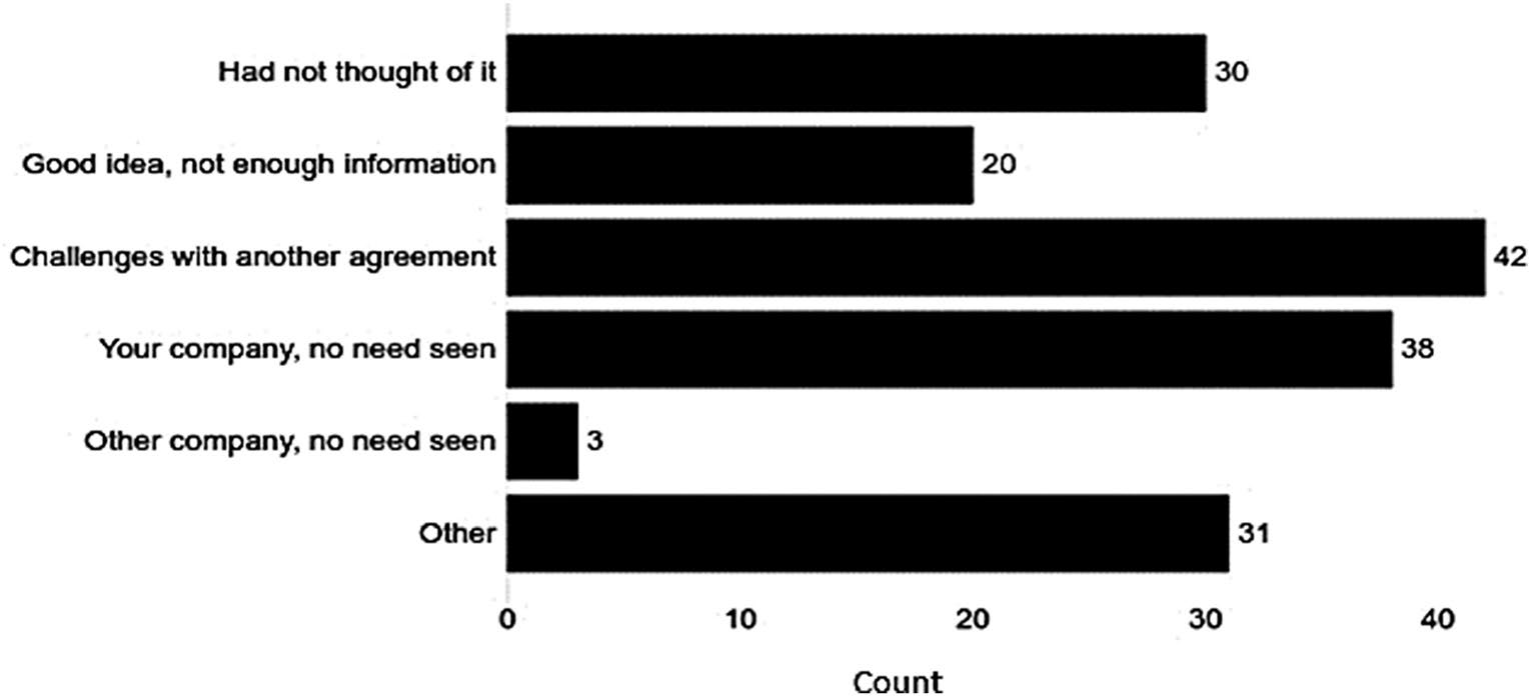
Reasons for Not Having Regulatory Agreements. What is the reason your company does not have standalone regulatory agreements? (number of responses = 164 of 191 respondents presented with this question)

Respondents in companies without standalone regulatory agreements were asked why these agreements were not in place. The challenge of having another standalone agreement was chosen most often (26%) from a list of 5 suggested reasons (Fig. 4). Alternatively, some saw no need for such an agreement (23%), had not thought of it (18%) or felt that it was a good idea but had insufficient information (12%). Rarely chosen was the reason that the partner company saw no need for such an agreement (2%). Other was selected by 19%; their comments were grouped in 4 themes that are listed under Fig. 4.

**Fig. 5:**
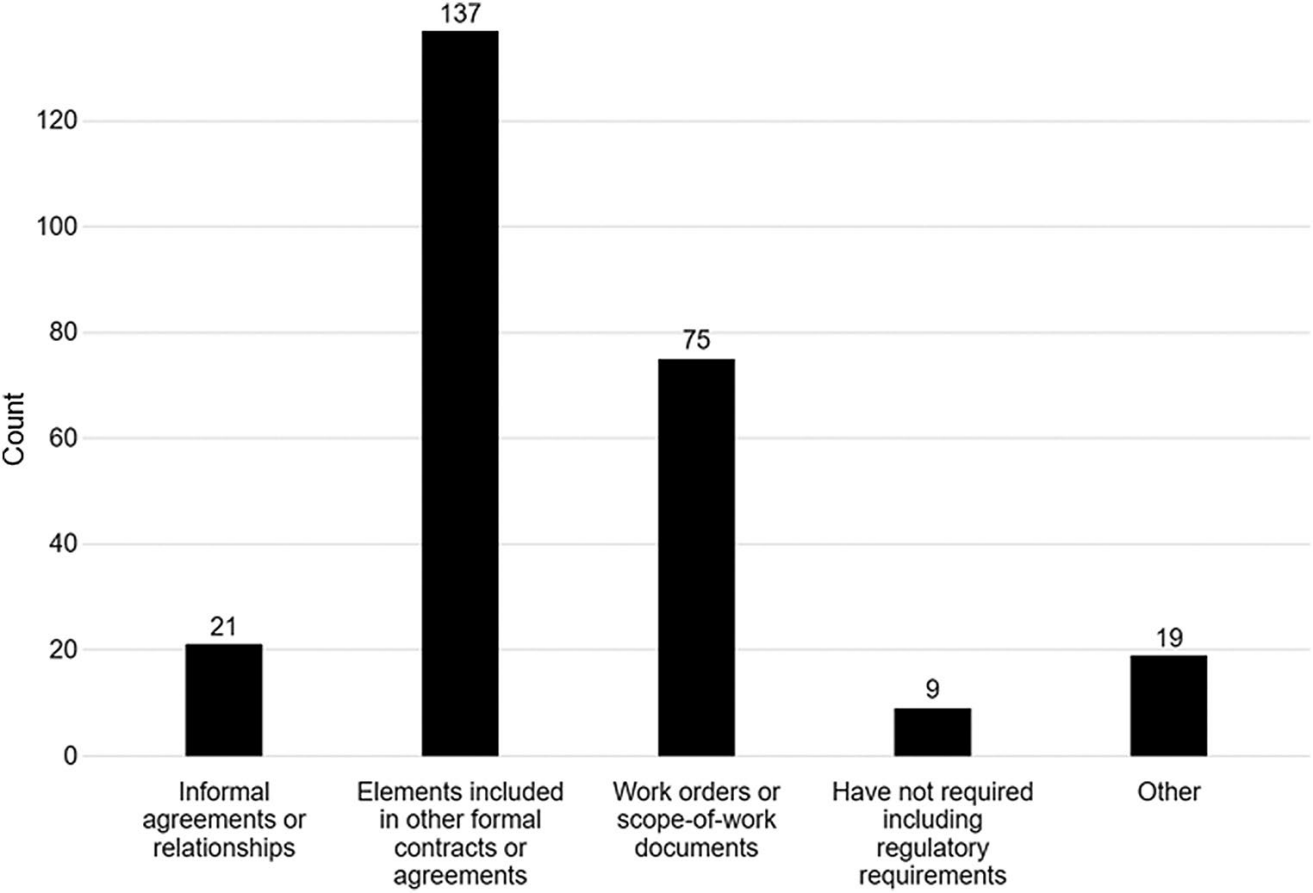
How Companies Without Regulatory Agreements Cover Regulatory Requirements with Their Partners. How does your company ensure that regulatory requirements are covered in their current partnerships with other companies and vendors? (choose all that apply) (number of responses = 261 from166 respondents presented with this question)

Asked how regulatory elements are covered currently in the absence of regulatory agreements, most respondents pointed to other formal contracts or agreements such as Master Service Agreements (MSA) (83%) (Fig. 5) or identified that they used work orders or scope-of-work documents instead (45%). A small group identified that they employed informal agreements or relationships (13%), or that they required no regulatory agreements in their partnerships (5%). Other comments (11%) expanded on the same categories that were included in Fig. 5.

**Fig. 6:**
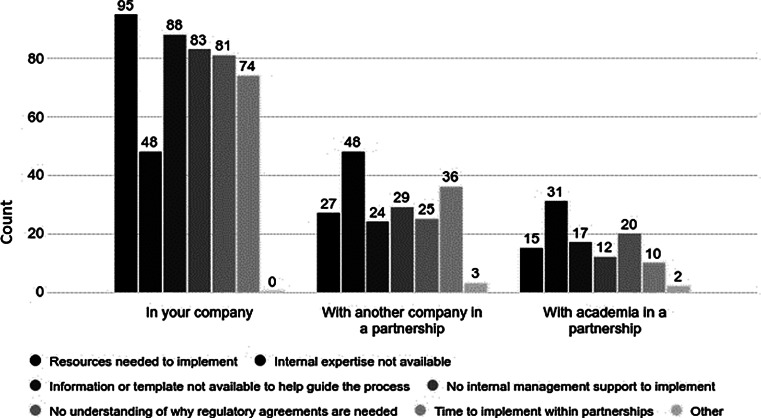
Impediments to Using Regulatory Agreements. What hurdles do you see in implementing the use of regulatory agreements with a business partner (pharmaceutical/biotechnology/device company) or an academic institution? (choose all that apply) (number of responses = 156 from 194 respondents presented with this question)

When asked about hurdles that they might anticipate if they were to implement regulatory agreements (Fig. 6), most predicted that the greatest hurdles would be faced within their own companies rather than those of their partners. These internal hurdles included: resource limitations (69%); lack of templates (68%); unsupportive management (67%); no recognition of a need for such documents (64%); and lack of time to set up such agreements (62%). Least often identified was lack of internal expertise (38%). However, a lack of internal expertise was identified as the biggest perceived hurdle for the partnering company (38%) or academia (20%).

### Additional Views from Respondents

All respondents were asked to rank 4 choices that might explain why we hear so little about the use of regulatory agreements (from 1 to 5, most to least likely) (Table 4). Most highly ranked was the choice that regulatory elements were covered in other contracts. However, closely ranked were: (2) the value of such agreements was unclear; and (3) the company had not thought about it. Least likely was the choice that informal relationships were sufficient (4). A few respondents offered other reasons, included in Table 4.

**Table 4:** Reasons Why Little is Heard About Regulatory Agreements. Why do you think we hear little about standalone regulatory agreements in the pharmaceutical industry? Please rank the following (1 being the most likely reason) by dragging and dropping each item. (number of responses = 225 from 294 respondents presented with this question)

Why Little is Heard About Regulatory Agreements	1 (most likely)	2	3	4	5 (least likely)	Rank Score^1^	Ranking
Value unclear	47	101	56	20	20	867	2
Had not thought about it	39	62	86	33	33	800	3
Covered in other contracts	130	38	46	9	9	967	1
Informal relationships are sufficient	4	21	32	156	156	668	4
Other	5	3	5	7	7	73	5
Ranking	Other Comments *n*=12						
1 (most likely)	• Companies may not be aware of regulatory agreements or have the time/resources to draft, review, revise, and then approve these agreements. • Confidential contract information is not disclosed to the public • Not required by regulators, unlike quality agreements • These are not legally required, and normally covered outside of contractual agreements, allowing some flexibility of management on both sides • do not know						
2	• 1/Not required by regulatory agencies. [sic] • Mostly, there is no need for a standalone regulatory agreement.						
3	• Companies equating a TORO document with a regulatory agreement. • No health authority guidance or mandate						
4	(No comments provided for this value)						
5	• Covered in the quality agreement • Focus on Service and / or quality agreements • Is not recognized as current best practice in industry						

^1^Rank score calculation described in Materials and Methods. Rank score calculated by applying a weight to each response. The number of responses ranked as 1 multiplied by 5, 2 was multiplied by 4, 3 by 3, 4 by 2, and 5 by 1. The total weighted score for each option was summed and divided by the number of respondents

### Proposed Agreement Framework

Most respondents with regulatory agreements suggested that their initial regulatory agreements included most of the basic development, review, and logistics elements provided as options in the survey questions but added additional logistical and management elements to strengthen the contract in later iterations as indicated in the comment fields above. These many elements can be used to develop a generic framework for a regulatory agreement between partners with a specific focus on agreements that involve shared responsibilities for regulatory submissions. Some of these elements would be inappropriate for agreements with other partners, and additional elements might be added to address activities unanticipated by the checklist provided in Table 5.

**Table 5:** Draft Checklist for a Regulatory Agreement

Category	Elements
Company and Organizational Information	Companies in this agreement • Contact information. Commitment to maintain qualified and knowledgeable regulatory and quality resources to support the commitments within this agreement, in case team members are fired, laid off, or leave the company
Product or Service	Product or service covered by the agreement
Regulatory Authorization and Compliance	Access and maintenance to required registrations, licenses and authorizations Compliance/conformity to established standards/guidance Oversight of GLP and GMP compliance Inclusion of shifting liability clauses Crisis management plan Penalties for non-compliance Database management and compliance Regulatory due diligence
Regulatory Documents and Records	Documents Process • Lead times, deliverable dates, turnaround times, expectations. • Management of authoring/review/modification/approval. • Number of review cycles Records management – • Handling • Ownership • Availability of source documents. Examples/Templates • Trial master file • TORO • Nonclinical study reports • Clinical study reports
Communications	Governance, decision making, and escalation processes Regulatory Communications • Lead times, deliverable dates, turnaround times, expectations • Responsibility for authoring/review/modification/approval • Responsibility for reporting responsibilities Company Communications • Information sharing plan Assumptions underlying the agreement Notification of audits, inspections, adverse (reportable) events
Roles and Responsibilities	Specific detail related to each party’s responsibilities Specific roles of individuals Sign off by wider range of functional areas for both parties. Specific roles and responsibilities of consultants Matrix defining accountabilities and responsibilities (RACI) Regional specific RA requirements, including R/R in each region Roles for crisis arrangements Definition of teamwork, process improvement, and mutual respect Consequences for lack of performance
Regulatory Submissions	Applications, Supplements and Amendments • Deliverable dates, turnaround times and expectations. • Responsibility for authoring/review/modification/approval. Labeling review and approval
Health Agency Meetings	Strategy for interactions Preparation of meeting materials Preparation for in-person or face-to-face meetings Attendance and roles/responsibilities for agency interactions (Written, telephone, and face-to-face)
Health Agency Requests	Responsibility and timing for authoring/review/modification/approval Reporting strategy
Regulatory Procedures	Regulatory strategy Labeling review and approval Postmarketing and lifecycle maintenance activities
Agreement Management	Template or decision tree to ensure completeness of the agreement Process/cycles for drafting and review of agreement Timing for review/modification of agreement: minimum of yearly or following major milestones: development, approval, and into the post market setting Clearer milestone agreements Process workflow diagram for collecting, reviewing, approving, storing and submission Audits of processes and records to verify compliance Process improvement Metrics • Deviations, CAPA and audit/inspection finding reviews. • Key Performance Indicators.
Publications	Rights and permissions to publish Timing of publications Authorship of publications

## Discussion

Today, medical product development depends on large teams of experts who contribute in different ways to business and regulatory decisions. The challenges of the COVID-19 pandemic have underlined the fragility of such relationships, as developers confronted challenges related to supply chains, working patterns and production needs [14–16]. Perhaps it was not surprising then, that half of the respondents surveyed here had heard about regulatory agreements, and a quarter, mostly from companies with more than 200 employees, had implemented them. In large companies, contractual relationships of many kinds are common, so developing standalone regulatory agreements is just one more way to establish a well-controlled development path shared between regulatory personnel in joint ventures when the responsibilities of two or more companies become deeply connected. Such a situation is common, for example, in biopharmaceutical companies, where one company develops a new drug but then partners with one or more larger companies to conduct clinical trials, manufacturing and eventual global commercialization.

This is not to say that such agreements should become a regulatory requirement. Rather they are a good solution for more complicated regulatory programs, as part of an armamentarium to clarify expectations between partners and meet regulatory requirements. These expectations include the US regulation, 21 CFR 312.52, *Transfer of obligations to a contract research organization*:

*A sponsor may transfer responsibility for any or all of the obligations set forth in this part to a contract research organization. Any such transfer shall be described in writing* [15].

The ICH Consensus Guideline, an addendum to the ICH E6(R1): Guideline for Good Clinical Practice ICH E6(R2), also includes the statement that:

*A sponsor may transfer any or all of the sponsor’s trial-related duties and functions to a CRO, but the ultimate responsibility for the quality and integrity of the trial data always resides with the sponsor. The CRO should implement quality assurance and quality control. (Sect. 5 Sponsor: Sect. 5.2.1)* [17].

However, the statements of regulatory authorities identify the sponsor’s ultimate responsibility for the conduct of the work. They do not specify how that requirement should be satisfied.

The stand-alone regulatory agreement has similar goals and structures when compared to its already well-accepted sister agreement, the quality agreement. FDA not only expects that companies will have quality agreements with key suppliers but has specified basic areas- documentation, change control, materials management, product specifications, and laboratory controls, for example- that such a document should address. Quality agreements should also clarify issues of compliance and communication between parties, which can be more challenging because the timing and depth of various work elements can change as time passes [18–20]. Agreements of these types usually serve as a starting point for defining the relationship but over time must be revisited to be sure that changing condition do not require a revision of terms.

The core value of a regulatory agreement is its promise to prevent miscommunication, unclear performance measures, wasted resources, and delays in decision making [21–23]. Regulatory agreements assume greater importance when the division of labor and extent of the collaborative activities become more complex. By covering a broad list of activities in one place, they help to avoid having a patchwork of items related to regulatory responsibilities in different documents, where they may be poorly visible to the affected regulatory personnel. They also provide an early opportunity to identify potentially non-compliant or unpromising partners. A key opportunity that regulatory agreements can offer is their ability to force the realignment of differing views regarding regulatory performance that might otherwise become contentious. However, not all potential issues are easy to capture even with a standalone agreement. These challenges relate to the more diffuse nature of the regulatory role, summarized by one respondent:

*The details of regulatory agreements may not be fully considered. This is due, at least in part, to softer deliverables associated with regulatory. With other parties, such as CDMO/CROs, deliverables are tangible and straightforward to define.*

Further, such agreements are less valuable if they lack enforcement clauses. Explicit statements are needed to specify corrective interventions or even legal actions that will be taken should the partner prove unreliable [24, 25].

Respondents made clear that stand-alone regulatory agreements are not the only way to manage relationships, especially when partnerships or contracted activities are simpler. It was therefore not surprising to find that that small companies are less familiar with such agreements and may in fact have less need for them if they are at earlier stages of maturity. It is important that any agreement be structured so that it does not hinder other aspects of the development process. It may be sufficient for early-stage companies to put a smaller set of regulatory requirements in manufacturing, quality or clinical agreements [1, 3, 8, 9, 26]. These regulatory requirements may be relevant to material sourcing and supply, authoring, review, and provision of quality documents needed for registration dossiers, or notification of regulatory personnel of audit findings or changes affecting the regulatory status of the facility [18, 27–30]. Master service agreements may also be a useful vehicle into which regulatory language can be added when one company carries the primary responsibility for most elements of the regulatory submissions and interactions.

Amongst the companies with regulatory agreements, approaches often appeared immature. The fact that most companies had no SOP in place for writing regulatory agreements may suggest that such agreements are not developed and managed consistently. Regulatory agreements might be used more often and more effectively if companies had better resources to guide their development. Although many respondents identified regulatory agency documents as potentially important sources of guidance, nearly one-quarter found that guidance on this topic was not helpful. Further, the usual sources of education on which regulatory professionals rely- trade meetings, government presentations, or consultants- fell short of their expectations. These results may point to ways in which governmental or professional organizations could help, by educating their membership about the use and value of such agreements. Further, companies could be helped if they had templates such as those already published for other types of vendor and partnership agreements [18, 28, 29, 31–33]. An internet search to find “templates for pharmaceutical regulatory agreements”, conducted April 11, 2023, produced no references to such documents. For this reason, we have attempted to provide a comprehensive checklist of items (Table 5) that might be used as a starting point for agreements that involve shared responsibilities for regulatory submissions. This basic framework can of course be adjusted as needed by the addition or deletion of elements to address unanticipated activities.

A key aspect of implementation is having sufficient resources and support [34], including the participation and support of internal stakeholders [35, 36]. Not surprisingly, some respondents were concerned that senior management and other relevant business functions were not engaged or supportive. Further, teams constructing regulatory agreements often failed to include members from relevant functions such as general administration and legal departments, commercial affairs, finance, business development and alliance management. The value of having a broadly based team has been emphasized for other types of agreements and might improve the acceptance of regulatory agreements at senior management levels. However, an unfocussed or untrained team could potentially impede the development of the agreement [37], so agreements would be easier to put into place if trained personnel could be dedicated to the construction of the regulatory agreement.

Survey validity in this study has depended on our ability to sample the target population appropriately. Survey participants were solicited widely from a specialized group of pharmaceutical and biotechnology professionals, mostly at the director level or above. This gave a lower response rate but also more freedom from bias than would be expected if the participants were invited only from known colleagues or contacts [38]. The response rate of 20% reported here is consistent with response rates seen in several recent studies conducted with busy executive respondents whose emails are often screened by company internet filters or administrative assistants [39–41]. Further, surveys with even a 10% response rate may be valid if those respondents are appropriate and able to provide rich data based on their knowledge [42]. Executives may also be wary of surveys because they fear that they will be asked to share company proprietary or protected information. Nonetheless, once the survey was started, professionals appeared to be strongly engaged, as reflected by their voluminous comments and completion rate of 83%.

## Conclusion

Regulatory agreements appear to be evolving in the same way as precedent agreements in manufacturing, quality, and clinical trials, but are used currently by only a subset of companies. They are not required in the US, have no current agency or industry templates and little agency guidance. The choice to use them appears instead to depend on the complexity of the relationship, the extent of management and cross-functional team support and the specific needs of the project. In place of standalone agreements, other companies have chosen to include regulatory elements in other types of existing agreements. These can be sufficient when contractual relationships are simpler but can leave out important issues that need to be defined when participating in complex and at times multi-partnered joint ventures. Regulatory agreements would seem to be judicious, then, for relationships in which the complicated regulatory activities must be shared between the partners. Whether and how to implement such agreements would seem easier to decide if companies had better tools and templates to develop such agreements. A framework based on the feedback of industry respondents was developed here to aid in this effort.

### Electronic Supplementary Material

Below is the link to the electronic supplementary material.

Supplementary Material 1
