# Supplementary figures and images for: Standalone Regulatory Agreements for Product-Development Collaborations in the Medical Products Industry
Source: Ther Innov Regul Sci. 2024 May 31;58(5):897–909. doi: 10.1007/s43441-024-00646-1 (PMC11335966; doi:10.1007/s43441-024-00646-1)

1. Survey


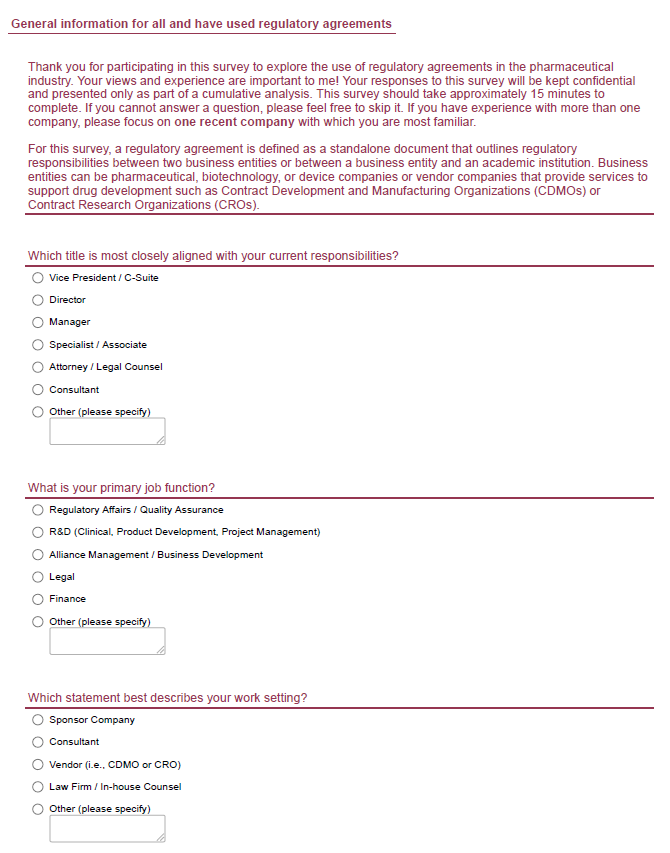


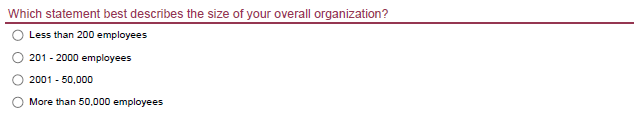


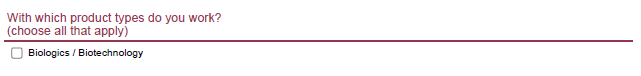


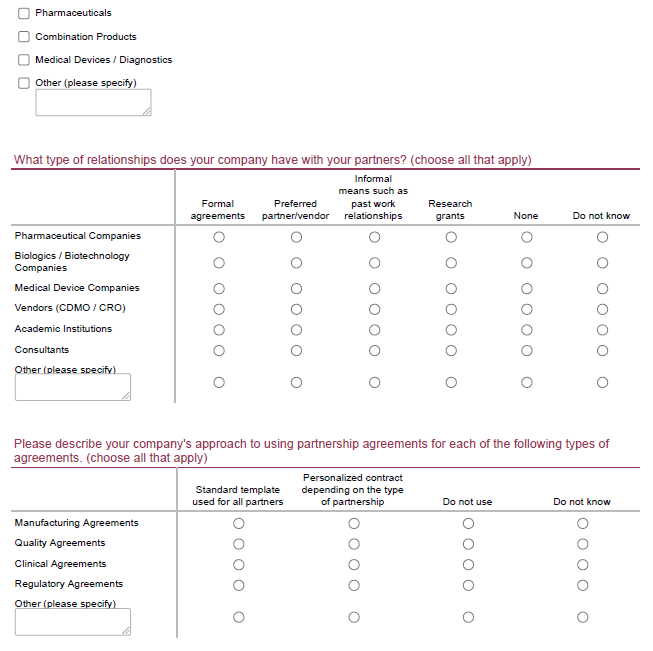


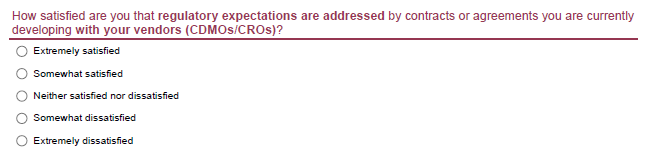


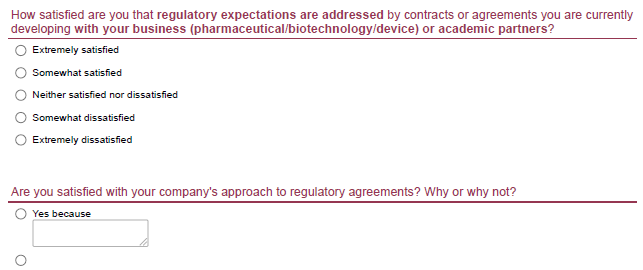


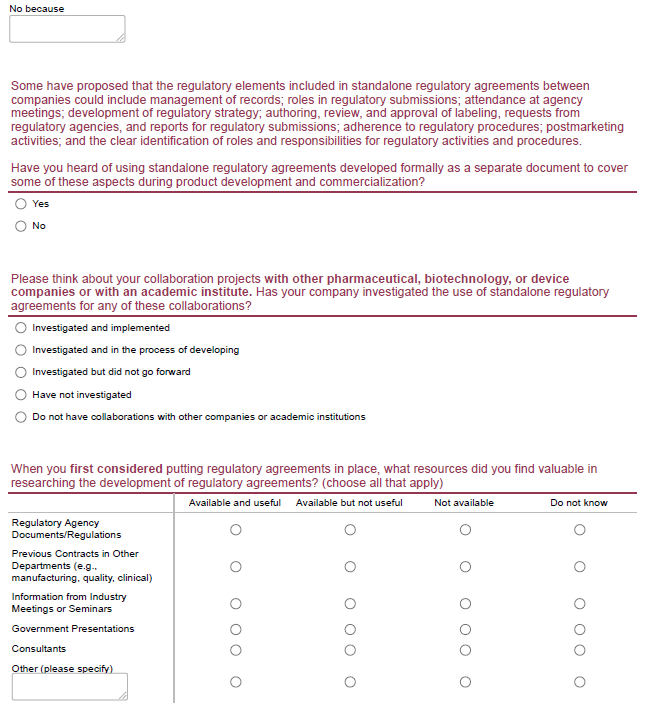


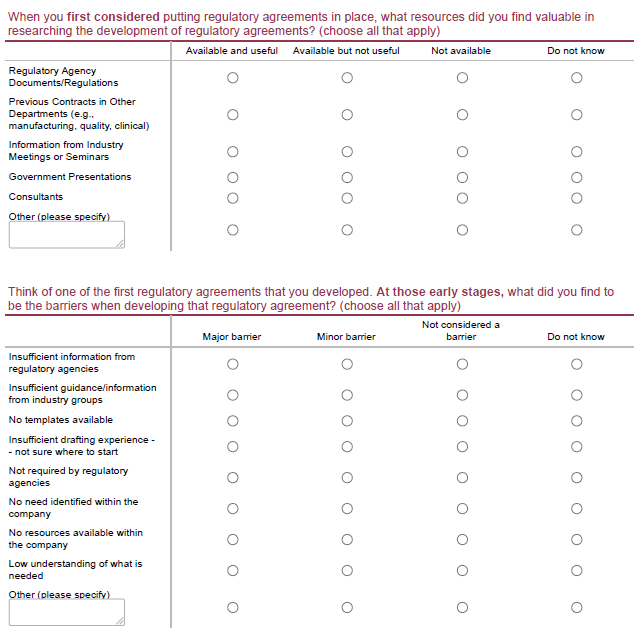


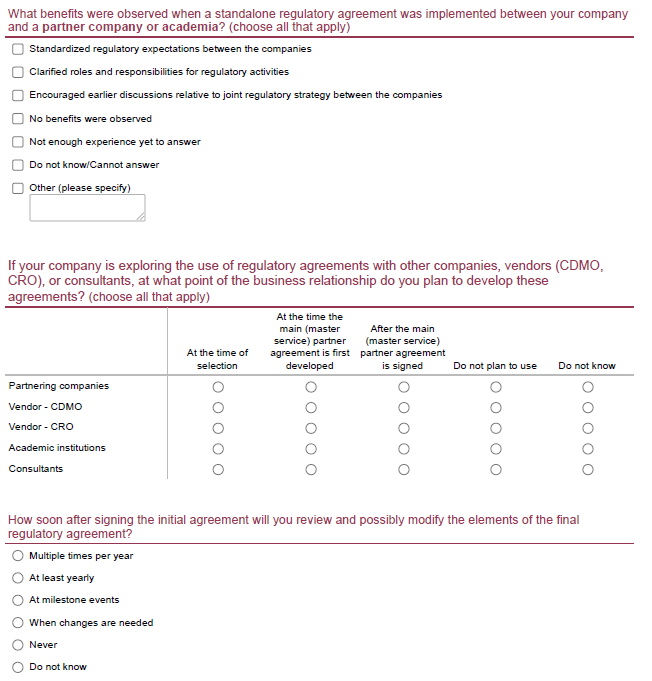


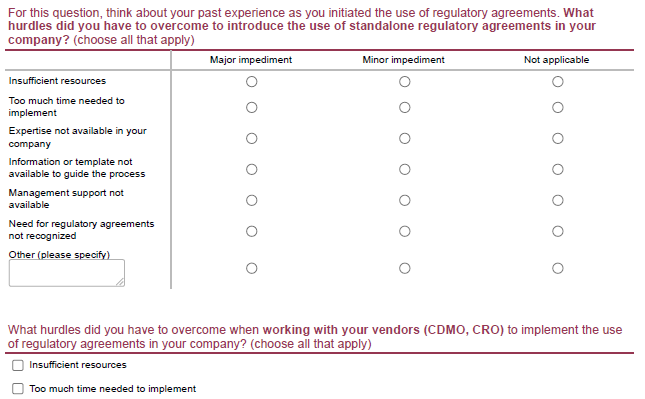


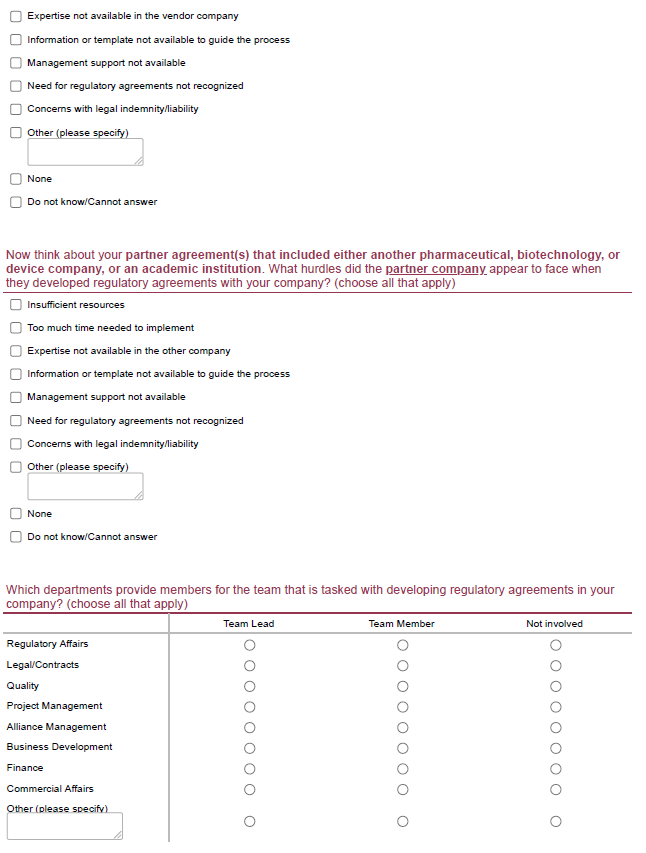


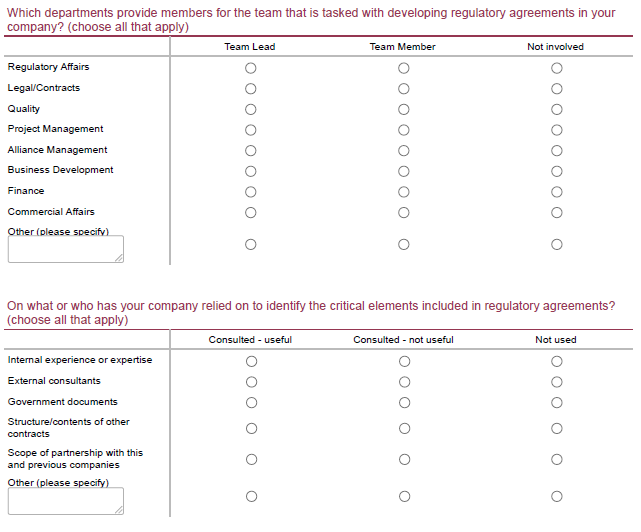


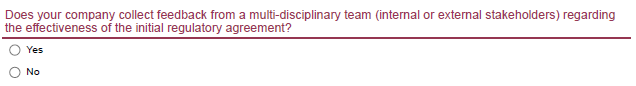


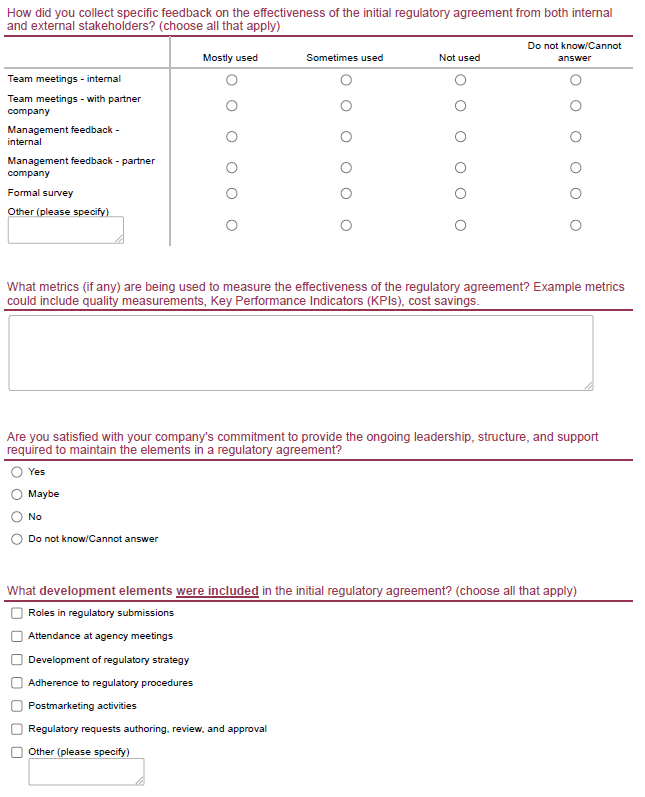


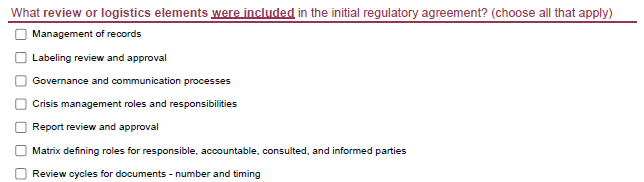


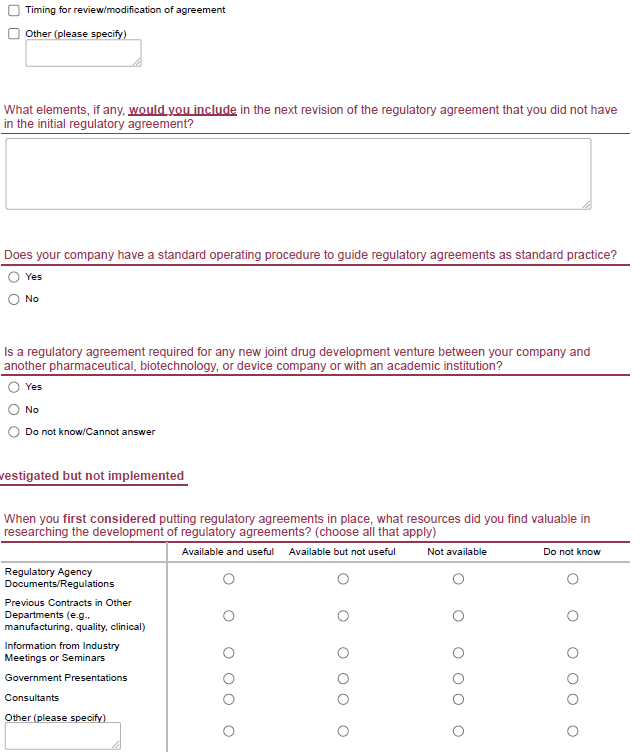


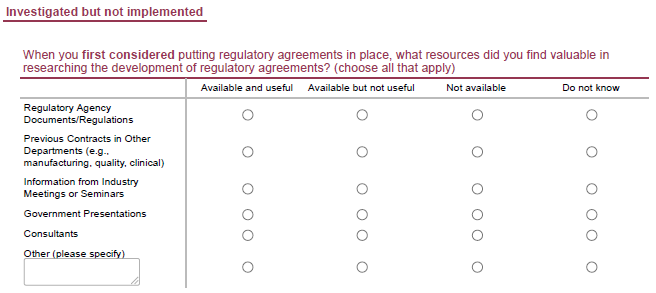


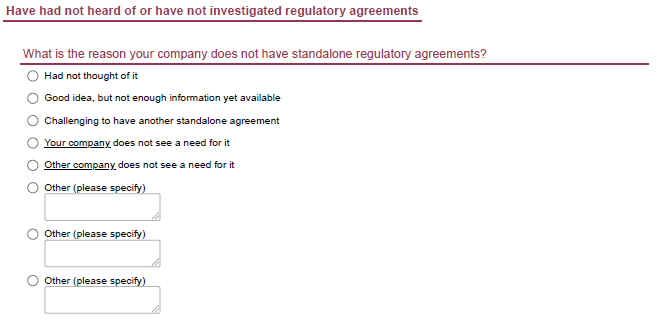


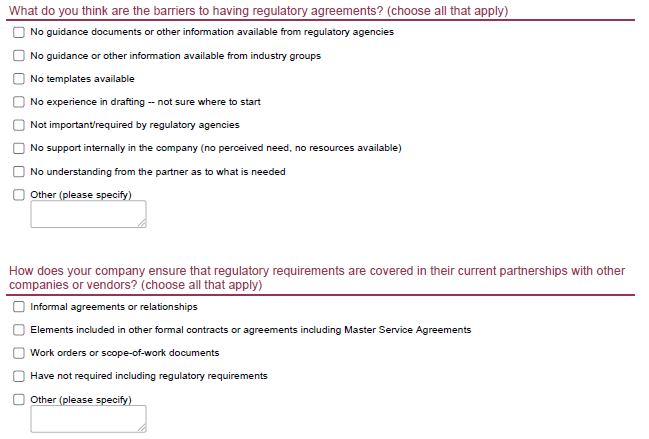


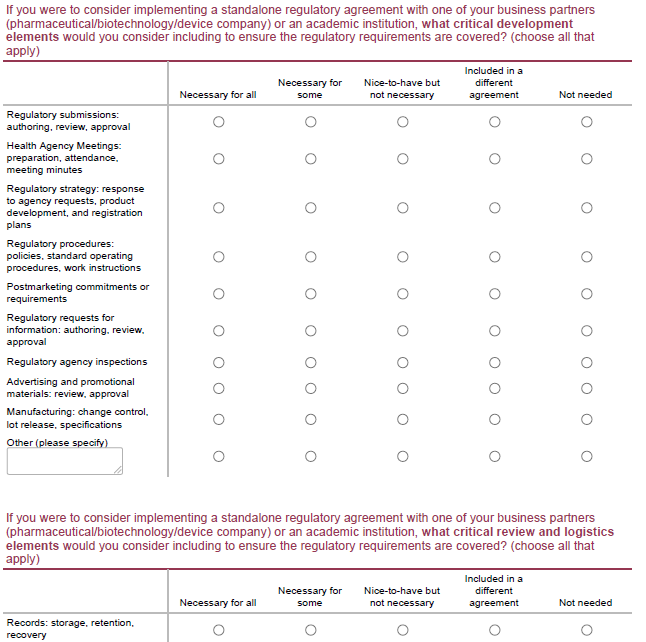


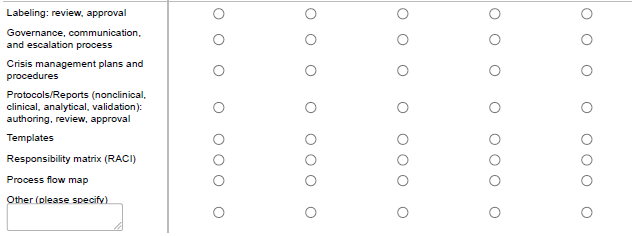


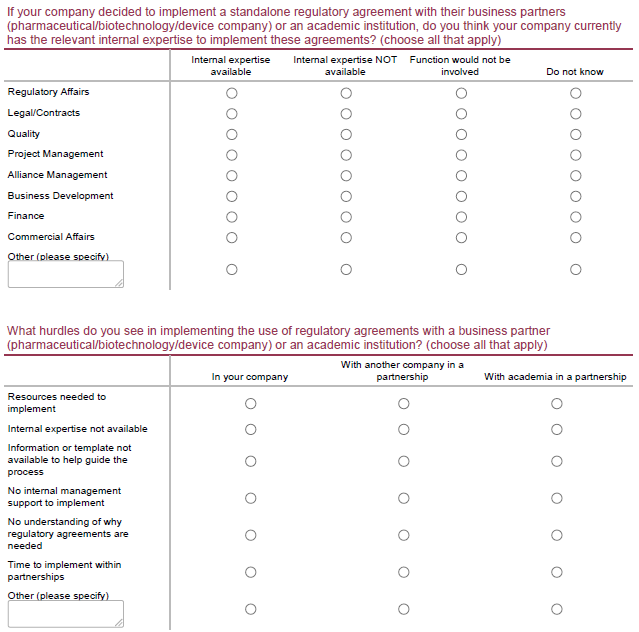


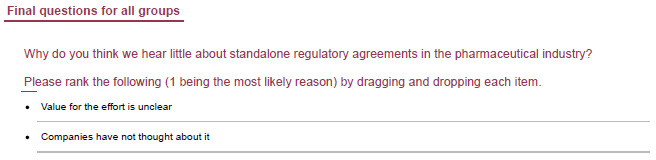


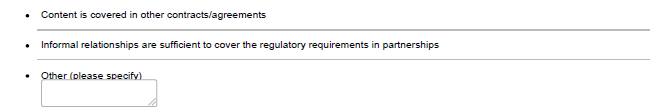


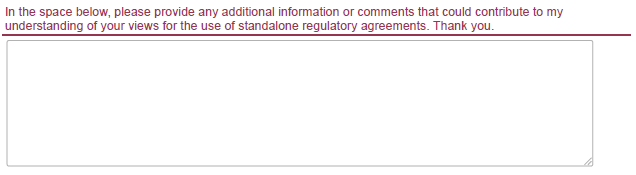

Supplement: Supplementary file 1 — Supplementary Material 1 [file 43441_2024_646_MOESM1_ESM.docx]
